# Removing mains interference from the mfERG by applying a post-processing digital notch filter: for the good or the bad?

**DOI:** 10.1007/s10633-021-09861-9

**Published:** 2021-11-30

**Authors:** Sven P. Heinrich

**Affiliations:** Eye Center, Medical Center – University of Freiburg, Faculty of Medicine, University of Freiburg, Killianstr. 5, 79106 Freiburg, Germany

**Keywords:** Multifocal electroretinogram, Mains interference, 50 Hz, 60 Hz, Notch filter, Noise, Spectrum

## Abstract

**Purpose:**

Ideally, the multifocal electroretinogram (mfERG) is recorded without noticeable intrusion of mains interference. However, sometimes contamination is difficult to avoid. A post-processing digital notch filter can help to recover the retinal response even in severe cases of mains interference. While a digital filter can be designed to have little to no impact on peak times, filtering out mains interference also removes the retinal signal content of the same frequency, which may result in a change of amplitude. The present study addressed this issue in the standard first order kernel mfERG.

**Methods:**

In 24 recordings from routine exams with no perceivable mains interference, the effects of 50-Hz and 60-Hz non-causal digital notch filters on amplitude and peak time were assessed. Furthermore, the effect of filtering on contaminated traces was demonstrated and simulated mains interference was used to provide an example of nonlinear superposition of retinal signal and mains interference.

**Results:**

mfERG amplitudes were reduced by 0%–15% (median 6%) with the 50-Hz filter and remained virtually unaffected with the 60-Hz filter. Simulations illustrate that spurious high-frequency components can occur in the filtered signal if a strongly contaminated signal is clipped due to a limited input range of the analog-to-digital converter.

**Conclusion:**

The application of a 50-Hz digital notch filter to mfERG traces causes a mild amplitude reduction which will not normally affect the clinical interpretation of the data. The situation is even more favorable with a 60-Hz digital notch filter. Caution is necessary if the assumption of linear additivity of retinal signal and mains interference is violated.

## Introduction

When recording a multifocal electroretinogram (mfERG), care should be taken to minimize the intrusion of mains interference [1]. However, tracking down the reasons for such interference can sometimes be challenging and time consuming. Occasionally, for instance with young children or with frail patients, delays in the examination procedure can be detrimental to the patient’s ability to cope with the measurement. In such cases, one might have to accept a suboptimal quality of the recorded data.

Excessive mains interference may be removed by means of a notch filter. Generally, filtering is considered to represent a useful technique for improving signal quality in electrophysiology [2, 3]. However, because the mfERG includes signal components at the same frequency as the mains interference, such a filter will not only eliminate the interference, but also affect the signal proper, as emphasized by the ISCEV mfERG standard [1]. Although alternative approaches have been proposed (e.g., by Fisher et al. [4]), a notch filter has the advantage of being very simple and easy to implement.

Actual empirical evidence for a substantial adverse effect of applying a notch filter to mfERG recordings or other retinal potentials is relatively scarce, though. Bock et al. [5] have demonstrated that the application of an analog 50-Hz notch filter can result a considerable reduction in amplitude with a marked increase in peak time in mfERG recordings. A study by Lachapelle and Molotchnikoff [6] showed amplitudes and the shape of the photopic full-field ERG response to be altered by a 60-Hz analog notch filter. In contrast, appropriate digital filters appear to have only little effect on the actual mfERG response, as demonstrated by Ledolter et al. [7] who applied an off-line 50-Hz notch filter to mfERGs recorded using a 2-global-flash stimulus sequence. Jingzhou et al. [8] applied an adaptive wave trap (details not provided) to eliminate simulated mains interference from an mfERG signal, and found the amplitudes and peak times to be mostly preserved.

The present article provides a quantitative and qualitative account of the effects of a post-processing digital notch filter on the first order kernel mfERG curves obtained with standard stimulation, i.e., the type of mfERG which is most often used in clinical applications. Importantly, while analog filters (or any ‘real-time’ filters applied at the time of data acquisition) cause a signal delay, this is not necessarily the case with post-processing digital filters as these can be designed to be ‘non-causal’ [9, 10]. This means, for instance, that peak times are preserved except for those changes that are directly related to the absence of that part of the signal which has been filtered out.

mfERG epochs are relatively short, typically in the order of one tenth of a second (*T* = 0.1 s). If the frequency spectrum of such an epoch is computed, the frequency resolution is relatively coarse (Δ*f* = 1/*T* = 10 Hz). This implies that eliminating one line of the frequency spectrum may have the potential of eliminating a considerable fraction of the signal. This could have an adverse effect on the mfERG, possibly reducing its ability to serve as a diagnostic tool.

**Fig. 1:**
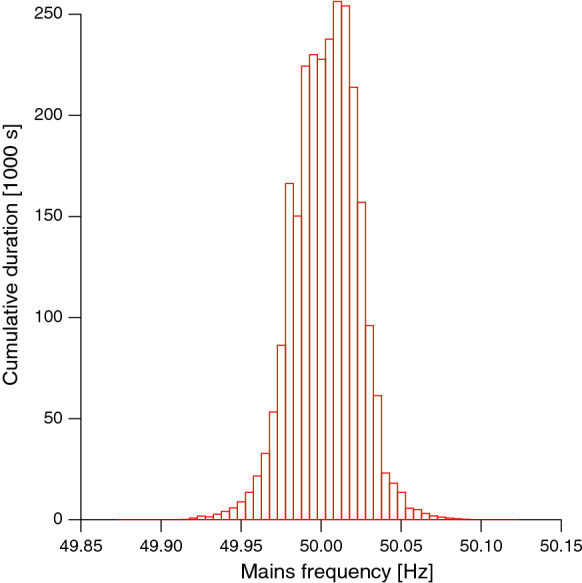
Histogram of actual mains frequencies for the full month of October 2020 as measured by the operator of our regional power grid, which is part of the synchronous grid of Continental Europe. Most of the time, the frequency is very stable within ± 0.05 Hz around the nominal frequency of 50 Hz. Only very rarely the deviation exceeds ± 0.10 Hz. Data source: https://www.transnetbw.de/de/strommarkt/systemdienstleistungen/regelenergie-bedarf-und-abruf

The effectiveness of a notch filter in reducing mains interference depends on the frequency of the interference being sufficiently stable. Although imbalance between use and generation of electricity within the electrical grid may cause fluctuations in mains frequency [11], these are typically very small (Fig. 1) and well below the coarse frequency resolution in mfERG filtering. Another possible reason for frequencies deviating from the nominal power line frequency is a variability in amplitude, which gives rise to ‘side bands’ in the frequency spectrum [12]. However, within the short epoch length, sizable fluctuations in amplitude are unlikely.

Higher harmonics may contaminate the signal if the mains interference is not perfectly sinusoidal. Even if higher harmonics are relatively low on the level of the regional electrical grid, local electrical equipment may introduce various types of distortions [13]. Higher harmonics could furthermore be introduced if the mains interference adds nonlinearly to the physiological signal, for instance due to amplifier nonlinearities or because the input range of the analog-to-digital converter is exceeded. Higher harmonics may be filtered out by extending the notch filter into a comb filter [14]. However, it is also possible to apply a low-pass filter to eliminate the respective signal components without adverse effect on the diagnostic value of the mfERG as shown by Han et al. [15]. The present article is therefore primarily concerned with the first harmonic.

## Methods

The present study re-analyzed existing mfERG data that were taken from routine examinations based on specific features as detailed in the results section. The use of the data had been approved by the local institutional review board. In addition to illustrating the effects of filtering by presenting qualitative examples, the present article also provides a quantitative account of amplitude (N1–P1) and peak time (P1) changes in a series of 24 routine mfERG recordings. About 20 of these were taken from 20 consecutive patients (one eye per patient) who met the inclusion criteria, starting at a random historical date. Primarily the central response was assessed. Inclusion of a patient’s eye was based on this response being not more than 50% below the lower limit of the lab’s normal range and not being perceivably contaminated by mains interference. If both eyes met the inclusion criteria, one eye was chosen at random. The other 4 recordings were specifically included to represent the upper range of amplitudes (above the median of the normal values), which was not well represented by the initial 20 patients. Because the study protocol as approved by the institutional review board prescribed the anonymized extraction of the mfERG data from the patient record, no further patient details are available.

All data had been acquired with dilated pupils using a VERIS Science 4.8 system and fiber electrodes (Ex-Stat 22/1, Statex GmbH, Bremen, Germany). Stimuli with 61 hexagons were presented on a FIMI Philips GD402/21CY9 CRT monitor with a frame rate of 75 Hz and a luminance of 500 cd/m^2^ for the white hexagons. For the present study, standard first order kernel mfERG curves were processed and filtered by applying the following steps.

1. Trimming the length of the mfERG curve to a multiple of 20 ms, which is the period of 50 Hz mains interference (or to a multiple of 16.67 ms in the case of 60 Hz). In our case, the resulting length is 100 ms. This ensures that the Fourier transform includes a spectral line exactly at 50 Hz and at 60 Hz.
2. Removing a linear trend in the data, defined by the first and the last point of the mfERG trace, in order to avoid introducing spurious spectral components [16].
3. Computing the discrete Fourier transform.
4. Zeroing the amplitude at 50 Hz or at 60 Hz. This represents a non-causal filter with zero phase-shift.
5. Computing the inverse discrete Fourier transform to reconstruct the mfERG curve.
6. Restoring the linear trend (cf. Point 2).

All analyses for the present study were performed with Igor Pro 8 (Wavemetrics, Inc.). Generally, the mains frequency in the present study was 50 Hz. However, the main quantitative analyses were also performed with a 60-Hz notch filter.^1^ Furthermore, a potential filtering artifact is demonstrated using an mfERG trace that has been artificially contaminated by adding a 50-Hz sine wave.

## Results

**Fig. 2:**
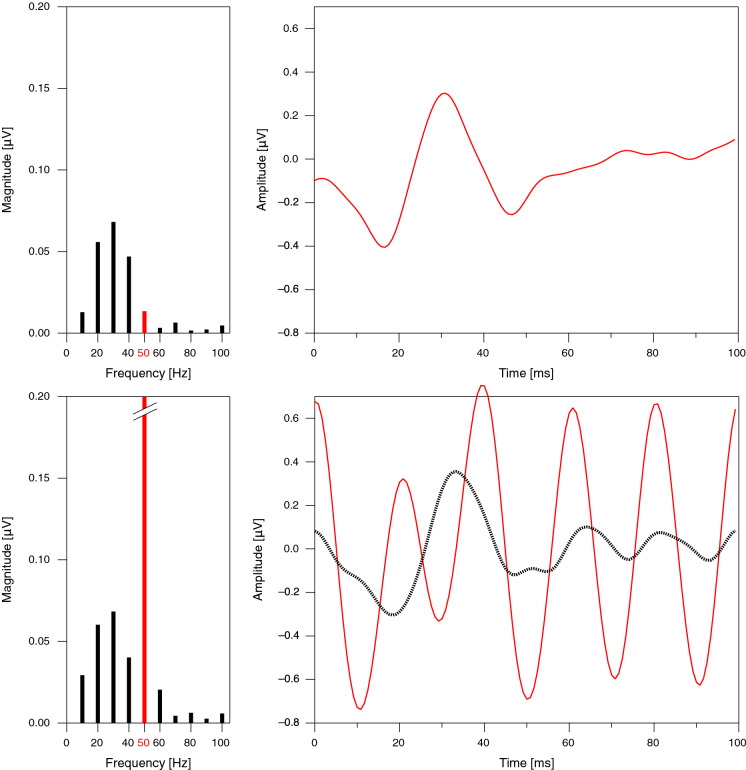
Top row. An mfERG trace with no perceivable mains interference (right) and the corresponding spectrum (left). Bottom row. MfERG traces with strong mains interference before (thin solid line) and after (dotted line) application of a notch filter. The spectrum (left) shows a large line at 50 Hz, which is removed by the filter

In all patients assessed for the present study, the dominant spectral components of the mfERG were in a lower frequency range than the mains frequency. An example is provided in Fig. 2 (top). Mains interference manifests itself as a strong increase in the 50-Hz spectral component (Fig. 2, bottom). Setting the 50-Hz line in the spectrum to zero eliminates the mains interference and reveals a typical mfERG response.

**Fig. 3:**
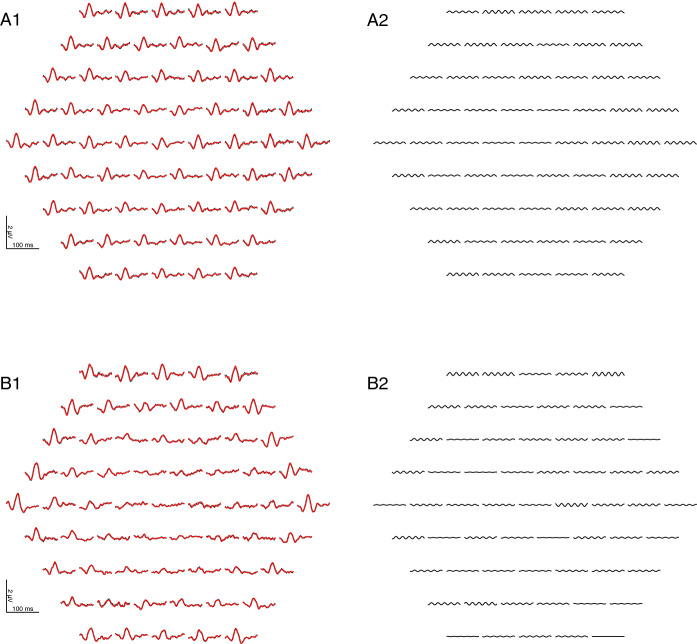
mfERG examples with a low level of mains interference. A1 and B1 show the measured (red) and filtered (black, dotted) mfERGs of two patients, one with a central response reduction. There is only little difference between the measured and the filtered curves, suggesting that filtering preserves the main characteristics of the mfERG curves. A2 and B2 show the respective 50-Hz components which have been removed by the filter

**Fig. 4:**
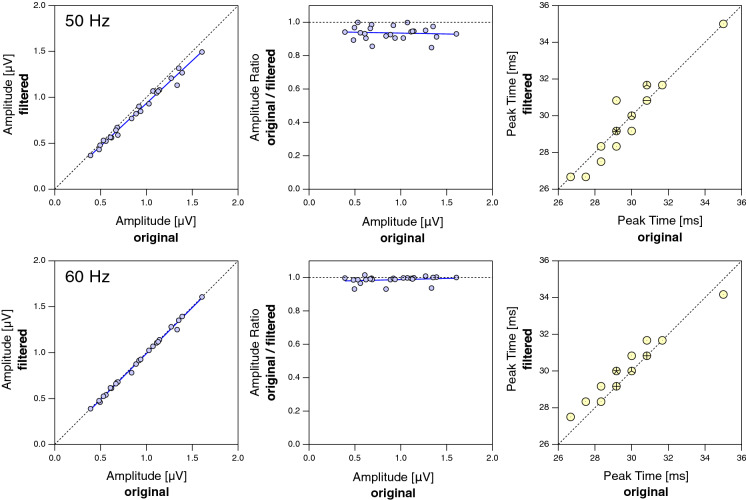
Top row, left: Effects of filtering with a 50-Hz notch filter on the individual amplitudes in a group of 24 patients. Amplitudes represent the N1–P1 difference. Amplitudes tend to be slightly smaller after filtering. The magnitude of this effect seems to increase with amplitude, as exploratorily corroborated by a trust-region Levenberg–Marquardt least orthogonal distance fit indicating a slope different from one (0.930 ± 0.024; blue line). Middle: The same data with the ratio of amplitudes (filtered/original) as a function of the original amplitude. The slope of the fitted line does not deviate substantially from zero (− 0.010611 µV^−1^ ± 0.0249 µV^−1^), suggesting that the filter effect is primarily proportional to the response amplitude. Right: Effect of filtering on peak times. With a single exception, the values remained unaffected by filtering or changed by no more than one sampling point (Δ*t* = 0.83 ms). Where several data points coincide at one location on the plot, these are collectively represented by a ‘sunflower’ marker [20] with the number of petals (sectors) indicating the number of data points. Bottom row: Same as top row, but with a 60-Hz notch filter. The effects, in particular concerning the amplitude, are virtually non-existent

Figure 3 illustrates for two sample recordings without noticeable contamination by mains interference that filtering does not have a sizable effect on the curve shape. This is confirmed by our sample of 24 patients (Fig. 4). The amplitude reduction caused by the 50-Hz notch filter ranged from 0.0% to 15.2% with a median of 6.0% (CI_95%_ (bootstrap): 4.5%…8.2%) without a sizable dependence on original amplitude. Peak times remained unchanged or shifted by usually not more than one sampling point (Δ*t* = 0.83 ms). With a 60-Hz filter, responses were virtually unaffected.

In order to ensure that filtering would not erroneously introduce an asymmetry between responses at temporal and nasal retinal locations, we compared the effects at the respective locations. A marginal difference was found for the 50-Hz filter (nasal amplitude reduction 6.7%, CI_95%_: 5.3%…8.0%; temporal amplitude reduction, 10.6%, CI_95%_: 9.4%…13.3%) while the 60-Hz filter hat no effect on amplitude at both locations.

**Fig. 5:**
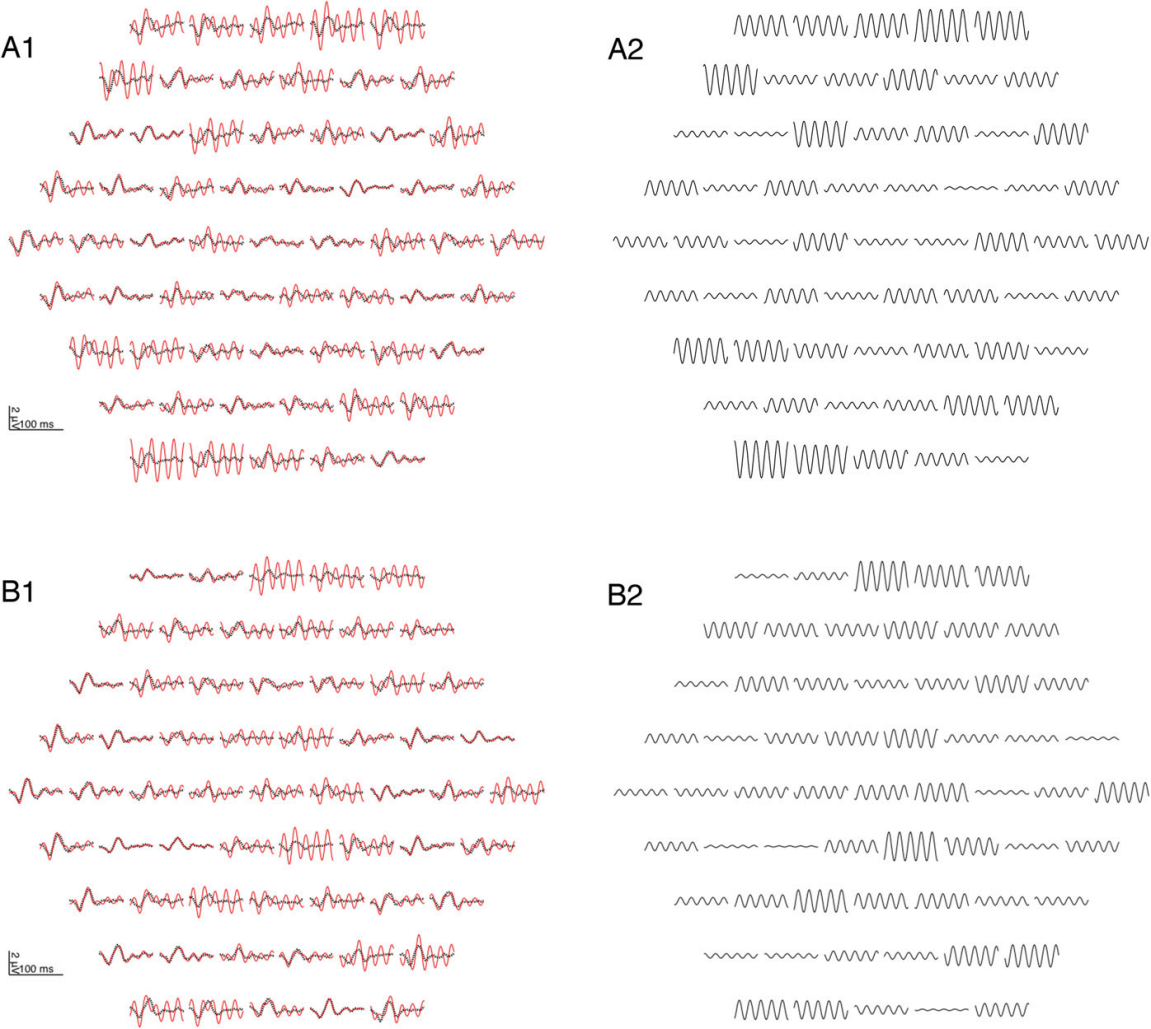
Two examples where sizable mains interference is present in a large subset of the traces. A1 and B1 show the measured mfERG (red) and the filtered curves (dotted black). A2 and B2 display the respective 50-Hz component. Many of the curves with a strong mains interference would be uninterpretable without filtering. Not all traces contain the same amount of mains interference because of constructive or destructive superposition effects which occur during the computation of the mfERG responses from the raw data

Figure 5 illustrates that strong contamination is reliably removed by the notch filter, while uncontaminated curves remain almost unchanged.

**Fig. 6:**
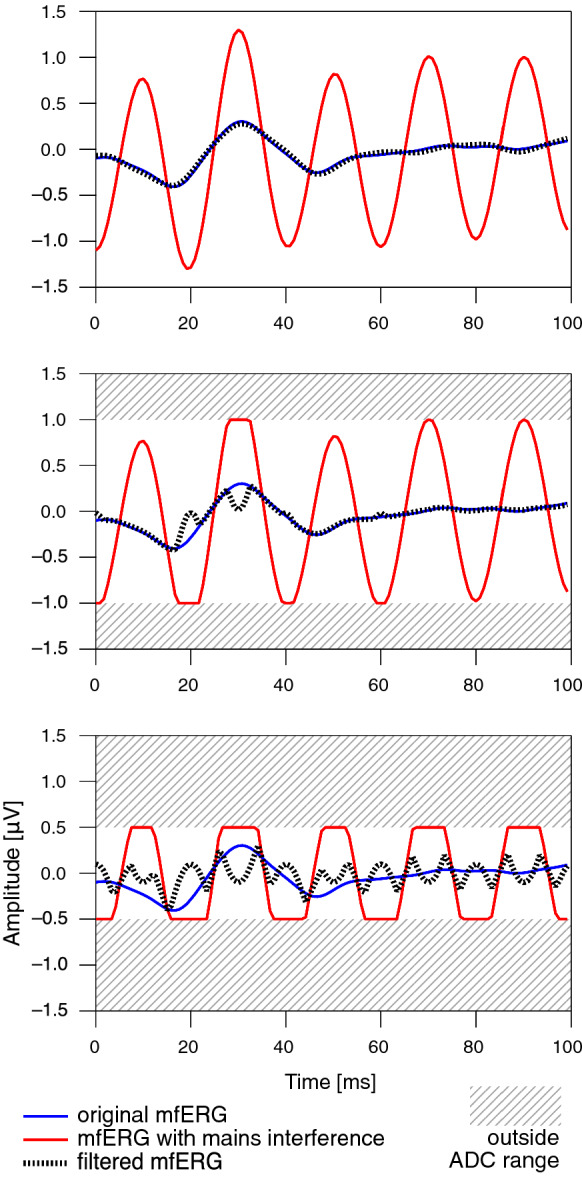
Effect of signal clipping. For this set of graphs, a low-interference original mfERG curve (blue) was superimposed with artificial mains interference (50-Hz sinusoidal curve with one of the maxima approximately aligned to the main positive deflection of the mfERG), yielding a signal with simulated contamination (red). The dotted black line shows the result of filtering. The top graph represents the situation where the full range of the signal is passed through the analog-to-digital converter (ADC). The filtered response reproduces the original curve almost exactly. In the middle graph, two maxima of the contaminated response were clipped, resulting in the signal exhibiting spurious fluctuations after filtering. If the input range of the ADC is even narrower, all maxima are clipped, resulting in a massive alteration of the filtered signal. The residual superimposed high-frequency components are essentially higher harmonics that contribute to the nearly rectangular curve shape. For display purposes, the simulation shown here assumes relatively narrow input ranges of the ADC. In reality, the input range is usually larger, and thus the problem manifests only at larger amplitudes of the mains interference

The data shown in Fig. 6 was created by superimposing an artificial 50-Hz signal onto a measured mfERG response. Three different assumed input ranges of an analog-to-digital converter are displayed. If parts of the signal are clipped due to the limited input range, this results in spurious response components.

## Discussion

It is not the intention of the present article to advocate bad recording techniques. ‘There is no substitute for good data’ (‘Hansen’s Axiom’, as cited by Luck [17]). Ideally, the recorded signals would have no perceivable mains interference. However, in those cases where mains interference cannot be avoided, filtering may be essential for making a recording interpretable. As shown above, applying a digital post-processing 50-Hz notch filter does not have a profound effect on the mfERG traces. In particular, there seems to be very little danger that the clinical interpretation of an mfERG would be substantially misguided by filter-related distortions of the mfERG traces. The alternative of leaving the traces contaminated by mains interference appears to be worse. The availability of reference ranges for filtered data would further facilitate the assessment of filtered patient recordings. Importantly, the 60-Hz filter has nearly no effect on the signal amplitude.

The median effect of 6% is lower than the mean relative intersession coefficient of variation reported by Meigen and Friedrich [18], who also used fiber electrodes. The tracking of subtle changes of mfERG amplitude over time could be further facilitated by submitting all recordings of a patient to a notch filter irrespective of the presence of mains interference.

Obviously, if a pathological condition were to alter the shape of a patient’s mfERG response such that a larger part of its spectral content would concentrate at the mains frequency, filtering would have a more detrimental effect. However, considering typical clinical cases, this concern appears more hypothetical than real. Normally, pathological signal changes, such as reduced amplitudes and increased peak times, would not cause the major frequency components to shift toward 50 Hz. This does not completely exclude small differential effects of filtering, for instance if responses from pathologically affected and unaffected parts of the visual field are compared within a patient.

It cannot be excluded that the amplitude reduction shown in Fig. 4 in the case of a 50-Hz notch filter partly reflects the removal of small amounts of mains interference that is invisible to the naked eye. However, the effect size appears to be approximately proportional to the response amplitude. This is expected for the respective retinal response component if the relative spectral composition of the response is independent of the amplitude. The marginal difference in filter effect between nasal and temporal locations should not be overrated given that the data was selected based on the characteristics of the central mfERG trace.

Before applying a notch filter to a massively contaminated recording, it seems worth to inspect the curves for features that would imply that the assumption of simple additivity of the mfERG proper and the mains interference is violated or that would result in a gross deviation of the latter from a sinusoidal shape. In particular, if the recorded signal (contaminated by mains interference) exceeds the input range of the analog-to-digital converter, adverse effects of filtering cannot be excluded. Caution is also necessary when interpreting recordings with large mains interference in the absence of any clear retinal responses, as this may be a sign of fundamental technical problems such as a broken electrode lead.

As long as the retinal signal and the interference signal superimpose linearly, the phase of the interference signal does not have an impact on the filtered signal. This is because the superposition (i.e., the sum) of two sinusoids of the same frequency is itself a sinusoid of exactly that frequency [19]. Therefore, filtering out the sum signal is equivalent to filtering out both constituent signals, namely the mains interference and the physiological signal component at the respective frequency, irrespective of their phases.

In summary, while it is clearly preferred to avoid mains interference during recording, a post-processing digital notch filter is a valuable tool in some situations and may greatly help to make contaminated mfERG data interpretable.

## Data Availability

Data are available from the author upon reasonable request.

## Footnotes

1 Note that the effect of a 60-Hz notch filter on the retinal response can be assessed despite the data having been collected in a region with 50-Hz mains frequency. The retinal response (approximated by recorded mfERG traces without noticeable mains interference) does not depend on the mains frequency.

## Declarations

### Statement on the welfare of animals

This article does not contain any studies with animals performed by the author.
